# Assessment of the Strength of Welded Joints in a Gas Pipeline Using FEM Modeling

**DOI:** 10.3390/ma19142959

**Published:** 2026-07-09

**Authors:** Olha Zvirko, Ihor Dzioba, Tadeusz Pała, Dmytro Demianchuk, Oleksandr Tsyrulnyk

**Affiliations:** 1 Department of Diagnostics of Materials Corrosion-Hydrogen Degradation, Karpenko Physico-Mechanical Institute of the National Academy of Sciences of Ukraine, 5 Naukova St., 79060 Lviv, Ukraine; dima.dem.fl@gmail.com (D.D.); otsyrulnyk@gmail.com (O.T.); 2 Department of Strength of Materials and Structural Diagnostics, Kielce University of Technology, Av. 1000-An. of Polish State, 7, 25-314 Kielce, Poland; pkmid@tu.kielce.pl; 3 Department of Machine Design, Faculty of Mechatronics and Mechanical Engineering, Kielce University of Technology, Av. 1000-An. of Polish State, 7, 25-314 Kielce, Poland; tadeusz.pala@gmail.com

**Keywords:** gas pipeline, welded joint, steel X52, microstructure, hardness, strength properties, FEM, modeling, mechanical properties

## Abstract

Repurposing existing natural gas pipelines for hydrogen service requires an assessment of their current technical condition and remaining lifetime, especially those that may have reached or exceeded their design life. In this study, the strength of welded joints in the X52 steel gas pipeline after 36 years of operation and in the as-received state was investigated, focusing on circumferential welded joints. Hardness distributions in the welded joint zones (the weld metal, the heat-affected zone and the base metal) were determined, and the values of the material’s strength and plasticity characteristics were estimated based on the correlation equations. This enables assessment of the stress–strain relationships for the metal from different zones of welded joints, which were compared with the experimental results determined from uniaxial tensile testing. Finite element models of welded joints were developed, in which the relevant material zones were defined in accordance with established stress–strain relationships, and the stress–strain distributions in the welded joints subjected to simulated tensile loading were determined, identifying the areas under the greatest stress. For the welded joint after 36 years of operation, the normalized graphs show higher relative extreme values of σ_11_/σ_ys_ = 1.25 compared to the as-received state, where σ_11_/σ_ys_ = 1.1. In the as-received welded joint, strain concentration was observed for a stress load about 5% lower than the σ_ys_ of the base metal, while in the operated one, a local strain increase was observed for a load even 11% lower than the σ_ys_ of the base metal.

## 1. Introduction

Pipelines play a key role in energy infrastructure by transporting large amounts of hydrocarbons over long distances. Their structural integrity and safe operation are of great importance because failures can lead to significant economic losses, environmental harm, and safety risks. Pipeline networks have been used intensively over the years, often exceeding their design lifetime. Each year, pipelines age further, while environmental requirements and awareness are increasing. Natural gas pipelines are designed mainly using low-alloyed steels, which are exposed to the influence of environmental factors and mechanical stresses. They operate under specific conditions. In addition to high pressure from the transported products, the pipelines are also affected by random dynamic and static loads. Random material defects can act as stress concentrators, and corrosive environments affect both the outer and inner surfaces of the pipe [1,2,3,4]. As a result, pipeline steels are degraded, which manifests in changes in the mechanical properties. Therefore, proper monitoring and timely assessment of such infrastructure is important for maintaining its structural integrity.

The degradation of pipeline materials during long-term operation is a complex, multifactorial process that depends on the material properties, environmental conditions, the type of loads, and the operational history. One of the most important reasons for pipeline damage is the combined effects of a corrosive environment and operational loads [5,6]. In the case of pipelines operated for a long time, there is an additional important aspect of changes in the mechanical properties, in particular a reduction in impact strength, fracture toughness, and changes in strength properties and plasticity with increasing time of operation [3,7,8,9,10]. General aging effects in pipelines include strain aging, dissipated damage accumulation, and deterioration of mechanical properties occurring over long-term service [3,7,8,9,10,11]. For X52 pipeline steel, service-induced changes are primarily associated with changes in brittle fracture resistance, strength and hardness [3,7,12]. Due to the corrosion of pipeline steels under operation with hydrogen evolution, including X52 pipeline steel, hydrogen can be accumulated in a metal, which promotes embrittlement and damage [3,13,14].

Among the various components of pipeline systems, welded joints (WJs) are generally considered the most critical locations due to the presence of metallurgical heterogeneities, geometric discontinuities, residual stresses, and potential welding defects that can act as stress concentrators and crack initiation sites [15,16,17]. Pipeline girth welds are subjected to complex loading conditions during manufacturing, installation, and service. These loads include internal pressure, bending, thermal stresses, residual welding stresses, and, in some cases, cyclic loading associated with pressure fluctuations. The welding process itself generates non-uniform heating and cooling cycles, leading to the formation of residual stresses and microstructural changes within the weld metal (WM) and the heat-affected zone (HAZ). These factors significantly influence the mechanical performance and fracture resistance of WJs [18,19]. Local variations in strength and hardness across the base metal (BM), HAZ, and WM can significantly influence the structural response of WJs [20,21,22].

The growing interest in hydrogen transportation through existing natural gas pipeline networks [11,23] has further increased the importance of assessing WJ integrity [24,25]. It is known [24,25,26,27,28,29,30] that weldments may exhibit increased susceptibility to hydrogen-assisted cracking and hydrogen embrittlement because of stress concentrations, heterogeneous microstructures, and residual stress fields generated during welding [26,27,28,29,30]. Numerous studies [26,27,28,29,30] have demonstrated that high-strength pipeline steels, such as X80 and X100, are generally more susceptible to hydrogen embrittlement than low- and medium-strength grades, including X42 and X52. Consistent with these findings, industry standards such as ASME B31.12 [31] and the CGA G-5.6 [32] recommend the use of low- and medium-strength pipeline steels for hydrogen transportation. Consequently, accurate prediction of the stress–strain state in WJs has become essential for evaluating the suitability of existing pipeline infrastructure for future hydrogen service.

The structural integrity of pipelines can be assessed through on-site, non-destructive mechanical and electrochemical testing using portable instruments [5,33,34,35,36,37,38,39,40,41,42]. This approach enables the early detection of material degradation and helps prevent unpredictable failures in pipelines. The indentation method induces only minimal disturbance to the stress–strain state of the metal and has attracted increasing attention in recent years for assessment of the actual mechanical properties of pipeline steels directly in service. Damage accumulation and aging processes in metals are primarily manifested through changes in their hardening behavior [5,43], which can be assessed by means of instrumented indentation and hardness testing [5,33,34,35,36,37,38,39,40,41,42].

Numerous researchers have applied finite element modeling to simulate welding processes and evaluate the structural performance of pipeline welds [44,45,46,47,48,49]. Advances in computational mechanics have made the finite element method (FEM) an indispensable tool for investigating the mechanical behavior of welded structures. FEM enables detailed analysis of stress distribution, plastic deformation, residual stress development, and crack-driving forces under realistic service conditions. Despite considerable progress in numerical modeling of pipeline welds, the accurate assessment of WJ strength remains a challenging task due to the combined effects of material nonlinearity, geometric complexity, residual stresses, and service loading conditions. Furthermore, many existing studies focus primarily on residual stress prediction or fracture behavior, whereas comprehensive evaluations of the stress–strain response of WJs under operational conditions remain limited. Studies involving gas pipelines that have been in operation for a long time, together with an assessment of the changes in mechanical properties, are particularly important. Reliable engineering diagnostics of gas pipelines is an urgent issue, the resolution of which would significantly reduce the material losses and the environmental and economic consequences caused by failures in gas transmission pipeline systems.

The accuracy and reliability of the results of strength analyses of welded components using FEM depend on various material and mechanical factors [20,21,22,45,46,47,48,49,50,51,52,53]. They are determined by the microstructural heterogeneity of the material, which leads to differences in the mechanical properties, and the influence of welding residual stresses. Moreover, FEM models can be affected by the correct selection of elements and boundary conditions. It is a very complex issue. Therefore, a simplified model (the BM and the WM) is often used, which gives conservative results [51,52,53].

The present study aims to assess the strength of WJs in the X52 steel gas pipelines after 36 years of operation and in the as-received state for comparison. The mechanical properties of the metal in various zones of the WJs based on hardness measurements and tensile tests have been investigated. Numerical tensile simulations of components containing WJs in the analyzed gas pipelines have been conducted using FEM to identify the weakest areas within them. The obtained results contribute to a better understanding of the mechanical behavior of pipeline welds and provide a basis for evaluating their suitability for long-term operation and future hydrogen transportation applications, as well as for the development of in situ non-destructive diagnostic procedures for long-distance pipelines.

## 2. Materials and Methods

The circumferential (girth) WJs used to connect pipeline sections for the transport of natural gas, made from low-carbon low-alloyed 0.17 C-1Mn-1Si pipeline steel (API 5L X52 strength grade), were investigated. This steel is commonly used in the construction of pipelines for the transport of gas or oil [54,55] and, according to API Specification 5L, has a yield strength *σ*_ys_ = 395–450 MPa and an ultimate tensile strength *σ*_uts_ = 528–560 MPa. Two sections of two pipes with WJs having an outer diameter of 1220 mm and a wall thickness of 12 mm were investigated: a pipe section with the field WJ had been in service for 36 years (operated state), and a pipe section with a WJ had been produced by the same technology for the reserved pipes (as-received state). In this study, the WJ in the as-received state is marked *WJ0*, and the 36-year operated one is *WJ36*.

**Figure 1:**
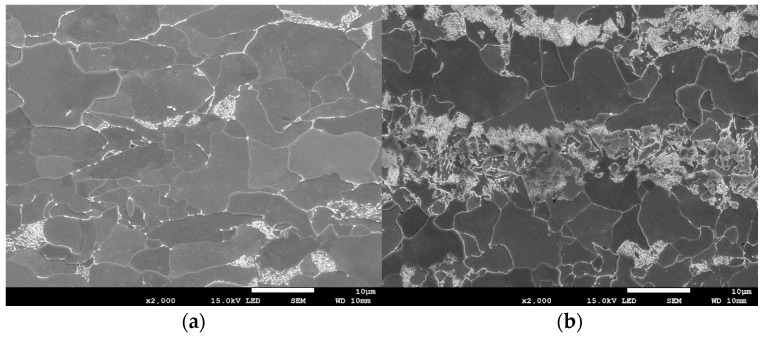
Microstructure of the BM in pipelines: (**a**) in the as-received state; (**b**) after 36 years of operation.

Metallographic studies of the microstructure of the BM were carried out using a JEOL JSM-7100F scanning electron microscopy (JEOL Ltd., Tokyo, Japan). The microstructure of the BM consists of ferrite-pearlite grains, typical of all carbon steels. However, as determined by metallographic examination, there are visible differences in the microstructure of the BM for the pipes in the as-received and operated states (Figure 1a,b). In the microstructure of the BM in the as-received state, the pearlite content is significantly lower than in the BM of the operated pipeline. These differences may be due to slight variations in the elemental composition of the steel and to specific production processes involved in pipe manufacture.

**Figure 2:**
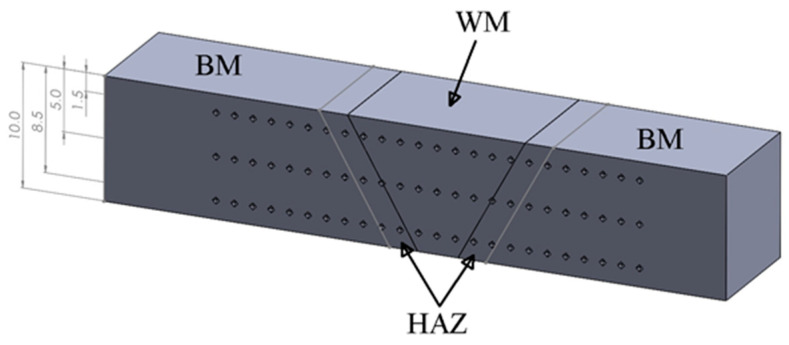
Scheme of hardness measurement in cross-section of WJ. Dimensions in mm.

Hardness measurements on the WJs analyzed were carried out on cross-sectional sections of the pipes, which were ground, polished and etched. The measurements were performed by the Vickers method (HV10) in accordance with the standard [56] using a Wolpert Wilson Instruments 310HVS hardness tester (Wolpert Wilson Instruments, Ludwigshafen, Germany). The hardness of the WJs was measured along lines situated at distances of 1.5 mm, 5.0 mm and 8.5 mm from the specimen surface, in zones covering the BM, the HAZ and the WM (Figure 2). The distance between individual indentations was 1 mm.

The strength and plasticity properties of the material in the individual zones of WJs were determined experimentally by a uniaxial tensile test in accordance with the guidelines of the standard [57]. Samples from individual zones of the WJs were cut parallel to the direction of the weld, after which cylindrical specimens with a diameter of 5 mm and a gauge length of 25 mm were machined. Uniaxial tensile tests were carried out on a Zwick-100 electrodynamic testing machine (ZwickRoell, Ulm, Germany), which is equipped with an automated control and data recording system.

Based on the tests carried out, nominal (engineering) tensile stress–strain curves were obtained, and the material’s strength and plasticity characteristics were determined using Equations (1) and (2). The nominal results from the tensile tests, taken within the range of uniform specimen strain (up to the point where the maximum load is reached and necking begins), were converted to values that account for changes in the specimen’s dimensions during the test (using Equations (3) and (4)). These values and the corresponding stress–strain relationships are hereinafter referred to as true.

(1) $$\varepsilon_{ni}=\frac{L_{i}-L_{0}}{L_{0}},$$

(2) $$\sigma_{ni}=\frac{P_{i}}{A_{0}},$$

(3) $$\varepsilon_{ri}=ln(1+\varepsilon_{n}),$$

(4) $$\sigma_{ri}=\sigma_{n}(1+\varepsilon_{n}),$$

where *L*_0_—initial gauge length of the specimen; *L*_i_—gauge length of the specimen during loading; *P*_i_—force applied to the specimen; *A*_0_—initial cross-sectional area of the specimen; *ε_ni_*—nominal strain (engineering); *σ_ni_*—nominal stress (engineering); *ε_ri_*—true strain; *σ_ri_*—true stress.

## 3. Results

### 3.1. Hardness Distributions

**Figure 3:**
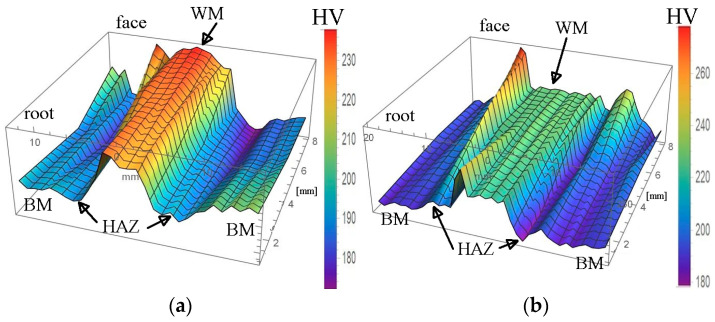
Map of hardness distributions of WJs: (**a**) *WJ0*; (**b**) *WJ36*.

In the joints on the face side, the WM has the greatest width, which gradually narrows to a minimum at the root. Examples of three-dimensional hardness distribution diagrams determined in cross-sections for WJs of the as-received state (*WJ0*) and operated (*WJ36*) gas pipeline are provided in Figure 3a and Figure 3b, respectively. These maps enabled the identification of local hardness gradients, heterogeneous regions, and transition zones between the BM, HAZ, and WM for the WJ depending on its state (as-received or operated).

**Figure 4:**
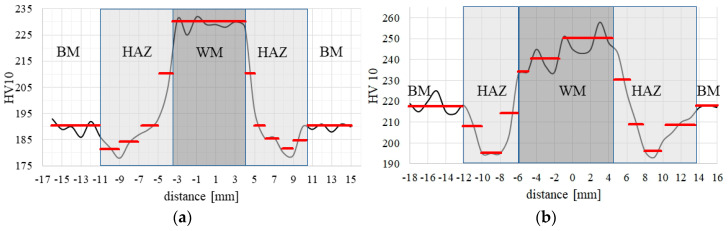
Selected hardness distributions of WJs determined along the measurement line located in the central part of the thickness of (**a**) *WJ0*; (**b**) *WJ36*.

Figure 4 shows the results of the hardness distribution along a measurement line located at the center of the WJs in terms of thickness. The results indicate significant variation in hardness, particularly in the HAZ. These graphs show the extent of the WM, the HAZ and the BM. The horizontal lines indicate the average hardness values for the respective zones and subzones of the joints, which were used in the subsequent analysis.

When analyzing the hardness distributions obtained for the investigated WJs (Figure 3 and Figure 4), it should be noted that the WM of both WJs has the highest hardness, followed by a decrease in hardness in the HAZ and a stabilization of hardness in the BM. For the as-received WJ, lower hardness values were also recorded for the individual zones compared to the operated one.

The hardness in the WM of the as-received WJ (Figure 3a and Figure 4a) is more uniform and equal to 225–233 HV10; then, it decreases to reach a local minimum of about 179 HV10 in the HAZ and stabilizes in the BM at the level of 185–194 HV10.

In the operated WJ, the WM has a hardness of 235–259 HV10; then, it gradually decreases with increasing distance from the weld axis to a minimum value of approximately 195 HV10 in the HAZ, finally reaching the values corresponding to the BM of 214–225 HV10 (Figure 3b and Figure 4b).

The higher hardness values in the BM and the HAZ of the *WJ36* joint compared to the *WJ0* joint are obviously due to the higher pearlite content in the steel.

### 3.2. Strength and Plasticity Characteristics

The presented hardness distributions (Figure 3 and Figure 4) show that the material properties in WJs are variable, namely, in the HAZ. Therefore, the cross-section of the WJ sample will contain materials with different properties, and the obtained results will reflect the interaction of layers of different materials and not a monomaterial. Therefore, to estimate the strength and plasticity characteristics in different zones of WJs, calculations were used based on previously developed correlation relationships describing the mechanical properties as a function of hardness [46,58,59,60,61,62].

These relationships have been derived from numerous studies [46,58,59,60,61,62] and are presented in the form of equations:

*σ*_uts_r_ [MPa] = 0.002 · (HV10)^2^ + 1.67 · (HV10) + 245.4; (5)

*σ*_ys_r_ [MPa] = −0.002 · (HV10)^2^ + 4.147 · (HV10) − 269.5; (6)

*ε*_uts_r_ [%] = 0.0002 · (HV10)^2^ − 0.155 ·(HV10) + 35.78; (7)

*ε* _ys_r_ [%] = −0.000003 · (HV10)^2^ + 0.003 · (HV10) − 0.152, (8)

where *σ*_ys_r_—stress corresponding to the yield strength value *σ*_ys_ calculated using Equation (4); *σ*_uts_r_—stress corresponding to the value *σ*_uts_ calculated using Equation (4); *ε*_ys_r_—strain corresponding to the yield strength value *σ*_ys_ calculated using Equation (3); *ε*_uts_r_—strain corresponding to the value *σ*_uts_ calculated using Equation (3).

**Figure 5:**
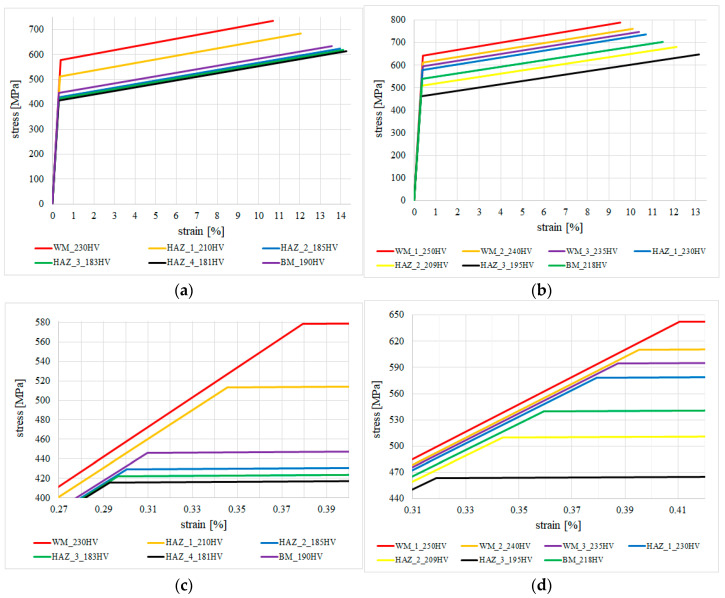
Stress–strain relationships and their magnifications at the yield strength of the material in the individual zones of WJs: (**a**,**c**) *WJ0*; (**b**,**d**) *WJ36*.

Knowledge of the material properties in different zones of WJs enables the development of stress–strain models, which are essential for numerical calculations during the simulation of loading on the component model. Figure 5a,b shows selected stress–strain relationships, which were estimated using correlation equations obtained from the hardness measurements presented in Figure 4a,b.

Table 1 and Table 2 present the data used to model the stress–strain relationships. The material models are presented as linearly elastic materials with linear hardening.

**Figure 6:**
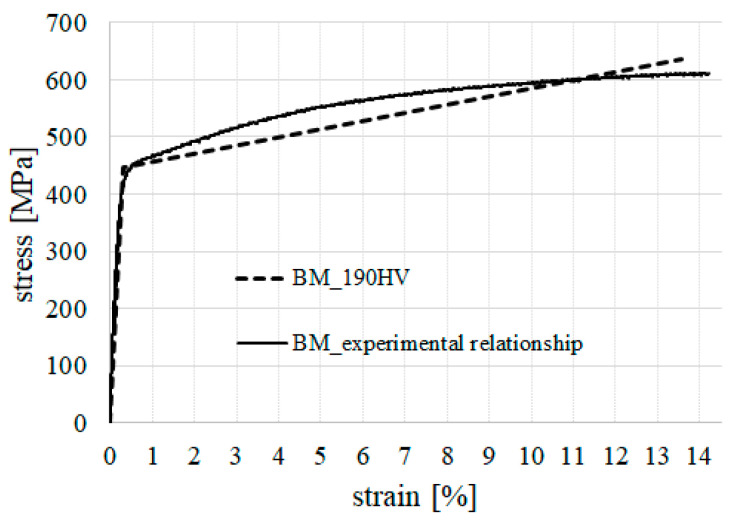
Comparison of experimental and modeled stress–strain diagrams for the BM of the pipeline in the as-received state.

**Figure 7:**
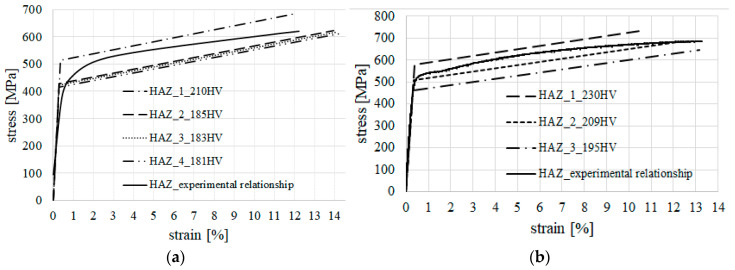
Comparison of experimental and modeled stress–strain diagrams of HAZ materials for welded joints: (**a**) *WJ0* and (**b**) *WJ36*.

The model stress–strain relationships were compared with those obtained experimentally during uniaxial tensile tests. Figure 6 and Figure 7 show selected diagrams for these relationships. In the case of the BM, which has a homogeneous microstructure, the theoretical and experimental stress–strain relationships are very similar (Figure 6). In contrast, the HAZ material is characterized by significant discrepancies between the experimental stress–strain relationship and the modeled ones for both WJs (Figure 7a,b). The variations in the model relationships are due to differences in the measured hardness values, which are a consequence of the heterogeneous microstructure in the HAZ, which is constantly changing [60,61,63,64]. Therefore, the cross-section of the specimen used in the experimental studies contains different types of microstructures, which leads to an averaged result.

Based on the results presented above, it can be concluded that the material properties obtained experimentally from specimens taken from the gas pipeline are comparable to those obtained using correlation equations. However, the use of correlation equations based on hardness measurements makes it possible to estimate the mechanical properties of the material and to establish material relationships in specific zones of welded joints.

### 3.3. Modeling of a Welded Joint

**Figure 8:**
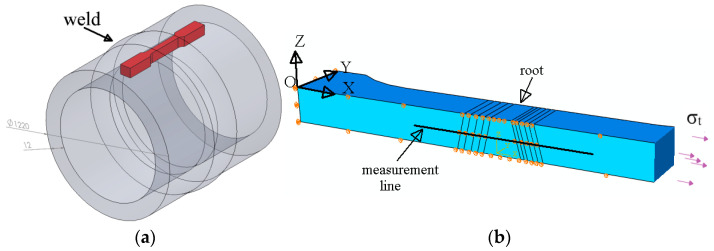
Gas pipeline with a circumferential WJ and the layout of the modeled specimen (**a**), and (**b**) a numerical model of the specimen. Dimensions in mm.

Knowledge of the strength and plasticity properties of the material used in welded joints was utilized to develop numerical models of the gas pipeline joints under analysis. The numerical model and load simulations were performed using ABAQUS 2017 version 6.18. Eight-node rectangular finite elements of type C3D8R were used in the modeling. A model of a section from a welded joint with a rectangular cross-section (dimensions 10 × 12.5 mm^2^) was developed. The location of the modeled section within the gas pipeline is shown in Figure 8a. The modeled element contains a weld with the HAZ and the BM on both sides. Due to the symmetry of the geometric shapes of the analyzed joints, half of the element was modeled. Displacements in the normal directions were blocked on the relevant XOZ and YOZ planes (Figure 8b). The joint models were simplified by omitting the weld face and root. This allowed for simplification, by reducing the number of finite elements, and could be completed at the same rate of calculation. An elastic–plastic material model with linear isotropic hardening was used for numerical calculations. Residual welding stresses were neglected in the FEM. The main limitations in the numerical modeling of WJs include difficulties in accounting for the actual joint geometry and assigning appropriate strength properties to individual zones, as well as determining the distribution of residual welding stresses.

The modeled WJ was subjected to simulated uniaxial tensile loading. In the numerical models, the load was generated by applying a stress value σ_t_ (Figure 8b).

Numerical simulations were carried out to evaluate the levels of strain and stress occurring in the material of individual zones of WJs in a gas pipeline when subjected to loads within the elastic–plastic deformation range.

**Figure 9:**
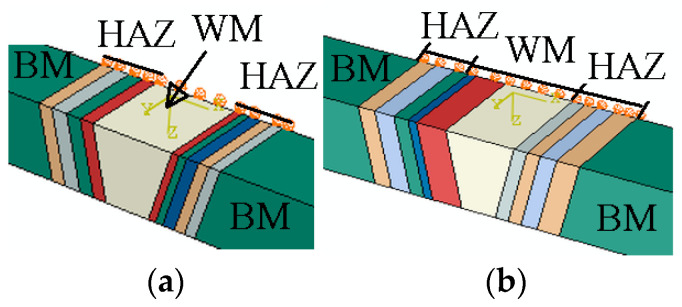
Distribution of zones in the model joints of the gas pipeline: (**a**) *WJ0*; (**b**) *WJ36*.

Based on the material characteristics data obtained from hardness measurements on the WJs in the model elements, zones with different properties were introduced. The location, dimensions, number, size and shape of the zones in the WJs were determined on the basis of the hardness distribution maps (Figure 3 and Figure 4). In the numerically modeled as-received WJ, six different ones were distinguished (Figure 9a), while in the operated WJ, seven zones were identified (Figure 9b). The subdivision of the HAZ for the as-received WJ and the HAZ and the WM for the operated WJ into multiple subzones was introduced to more accurately represent the variations in the mechanical properties observed by the hardness tests. For each material zone, the corresponding characteristic stress–strain relationship has been derived. Specific material properties were assigned to individual zones in the WJs of the gas pipeline, both in the operated and as-received state, which were obtained on the basis of functional relationships describing the mechanical properties and hardness. Residual welding stresses were neglected in the FEM.

Numerical simulations of the tensile behavior of the WJs made it possible to assess the levels of stress and strain arising within them. On this basis, it enables predicting the areas where the material is most stressed and where the joint damage process may develop.

**Figure 10:**
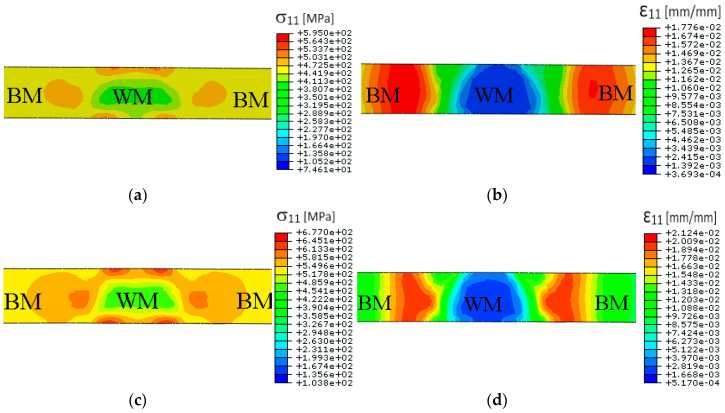
Stress (**a**,**c**) and strain (**b**,**d**) maps in loaded elements of WJs: (**a**,**b**) *WJ0* (σ_t_ = 460 MPa) and (**c**,**d**) *WJ36* (σ_t_ = 550 MPa).

**Figure 11:**
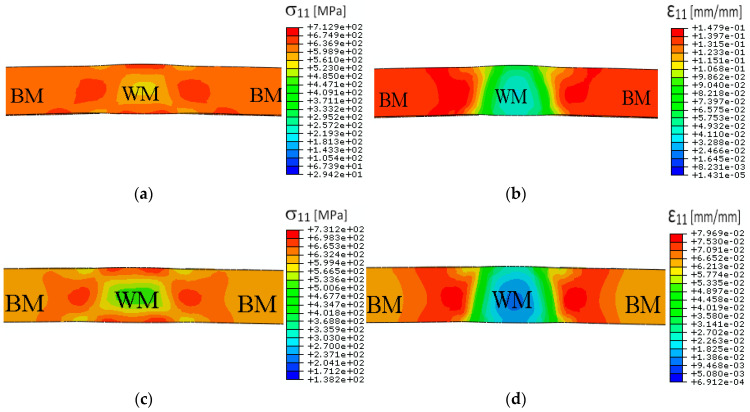
Stress (**a**,**c**) and strain (**b**,**d**) maps in loaded elements of WJs: (**a**,**b**) *WJ0* (σ_t_ = 620 MPa) and (**c**,**d**) *WJ36* (σ_t_ = 620 MPa).

The WJs were loaded with tensile stress corresponding to the direction along the pipe. The analysis presents two load levels—one corresponding to and the other exceeding the yield strength of the pipe’s BM. Figure 10 and Figure 11 show maps of the stress and strain distributions in the plane of symmetry of the specimen. This plane is located inside the specimen; therefore, the mechanical fields displayed on it are similar to those on the axial planes within the pipe material.

In the case of loading the WJs with a stress equal to the yield strength value (σ_t_ = σ_ys_) of the BM (Figure 10), higher stress levels were recorded in the HAZ, and they were lower in the WM compared to the BM. In the HAZ, the increase in stress in relation to the load level is insignificant—approximately σ_11_ = 1.04∙σ_t_ for *WJ0* and σ_11_ = 1.07∙σ_t_ for *WJ36*. However, the reduction in the stress in the WM is more pronounced—approximately σ_11_ = 0.87∙σ_t_ for *WJ0* and σ_11_ = 0.83 σ_t_ for *WJ36* from the load value. Accordingly, in the HAZ, strains of σ_11_ = 0.018 for *WJ0* and σ_11_ = 0.021 for *WJ36* were recorded, indicating the presence of permanent strain in the material. The strain levels in the WM are low, corresponding to the elastic range.

With the increase in the load to σ_t_ = 620 MPa, changes in the distributions of the stress and strain fields were observed (Figure 11). For the BM of the *WJ0*, the load value σ_t_ = 620 MPa is equal to σ_t_ = 1.39∙σ_ys_, while for *WJ36*, σ_t_ = 1.15∙σ_ys_. Therefore, the changes in mechanical fields in *WJ36* are less intense compared to *WJ0*.

In the operated WJ36 joint (Figure 11b,d) loaded with σ_t_ = 620 MPa = 1.15∙σ_ys_, the stress and strain distributions are similar to those observed at σ_t_ = 550 MPa = 1.02∙σ_ys_. The maximum values of σ_11_ = 670 MPa occur in the HAZ near the BM, which is 1.08 times higher than the load stress level. In the WM, the stress level is approximately σ_11_ = 520 MPa, which is below the load stress (σ_11_ = 0.84∙σ_t_). Maximum strains were also observed in the HAZ (ε_11_ = 0.08). The strain level in the WM is not high and is rather in the elastic range (ε_11_ = 0.0007–0.008).

In the as-received *WJ0* joint (Figure 11a,c), the loading stress σ_t_ = 620 MPa significantly exceeds the yield strength of the BM, σ_t_ = 1.39∙σ_ys_. This causes high levels of stress and strain throughout the entire measuring section of the specimen. The maximum values of σ_11_ = 650 MPa (σ_11_ ≈ 1.05∙σ_t_) were also observed in the HAZ in this joint. In the WM, the stress level decreases to σ_11_ ≈ 560 MPa, which is lower than the loading stress (σ_11_ ≈ 0.9∙σ_t_). The highest strain levels occur in the HAZ and reach values of ε_11_ = 0.14–0.145, which means high plasticization of the material in this zone. High strain levels were also observed in the BM: ε_11_ = 0.12–0.125. The lowest strain level is in the WM: ε_11_ = 0.03–0.045.

**Figure 12:**
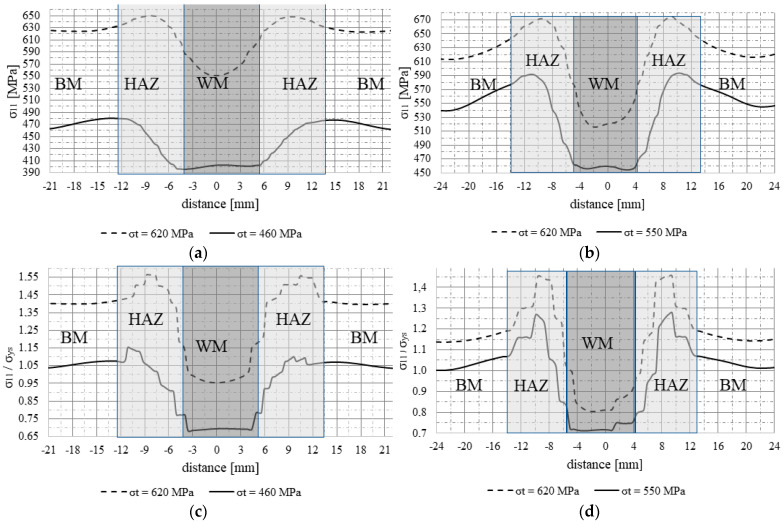
Stress distributions along the center line (axis) for (**a**,**c**) WJ0 and (**b**,**d**) *WJ36* joints: (**a**,**b**) nominal values; (**c**,**d**) values normalized by the yield strength.

The results of numerical tests of the stress distribution in the form of graphs along the center line of the analyzed element cross-section are presented in Figure 12. In Figure 12a,b, the graphs were prepared on the basis of the nominal stress values σ_11_ (in the tensile direction) obtained from the FEM analysis. The stress distributions in the graphs (for two load levels) properly confirm the analysis presented based on the stress fields described above. As for the load value equal to the yield strength level (continuous line), as well as for the higher level (dashed line), the maximum stresses σ_11_ occur in the HAZ area on the side of the BM. However, in the WM, a significant reduction in the level of stresses σ_11_ was observed. For both tested joints *WJ0* and *WJ36*, qualitatively similar stress distributions were obtained. Some differences in the level of the obtained values result from the use of steel with different microstructures and levels of mechanical characteristics for the production of pipes.

Figure 12c,d shows the above distributions normalized by the yield strength values of the material from the corresponding WJ zones. In this interpretation, the areas of the most stressed material in the WJs are more clearly visible: these are the HAZ on the side of the BM for both WJs. For *WJ36*, the normalized graphs show higher relative extreme values of σ_11_/σ_ys_ = 1.25 compared to *WJ0*, where σ_11_/σ_ys_ = 1.1.

**Figure 13:**
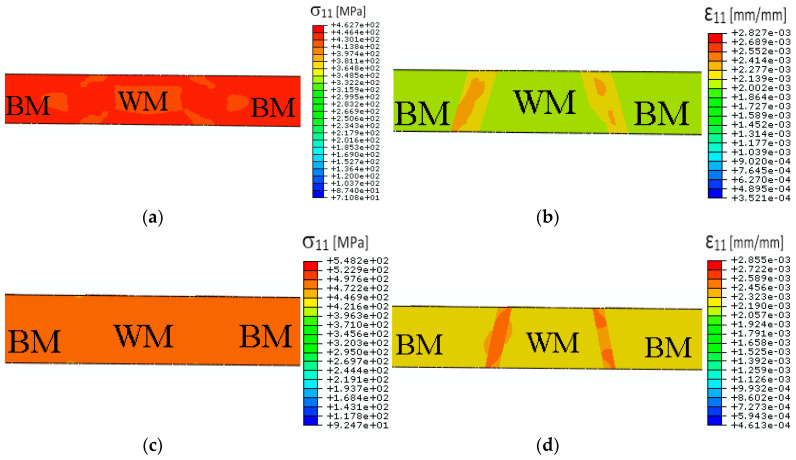
Stress (**a**,**c**) and strain (**b**,**d**) maps in loaded elements of WJs (**a**,**b**) *WJ0* (σ_t_ = 430 MPa) and (**c**,**d**) *WJ36* (σ_t_ = 480 MPa).

In the tested elements loaded to a stress level slightly below the yield strength of the BM, a local increase in strain was also observed in the HAZ near the BM (Figure 13). In the WJ0 joint, strain concentration was observed for a load 5% lower than σ_ys_ of the BM, while in the *WJ36* joint, a local strain increase was observed for a load even 11% lower than the σ_ys_ of the BM. At this point, the differences in the stress map distributions are almost imperceptible. When the element load was subsequently reduced by a few percent, no differences were observed in the mechanical field maps at all.

## 4. Discussion of the Obtained Results

The research results of the WJs of two gas pipelines made of the X52 steel, one in the as-received state and one after operation for 36 years, indicate that the BM of the operated pipeline has a higher hardness, yield strength and ultimate strength compared to the as-received one. The main objective of this study was to analyze the strength of these joints. The assessment was based on the results obtained from a numerical simulation of tensile loading on the modeled joints. The results, in the form of stress and strain distributions in individual zones, made it possible to identify the most stressed areas in the WJs. For the numerical modeling of the WJ, the HAZ requires special attention due to its heterogeneity, resulting in significant differences in the mechanical properties in its subzones [65,66]. The experimental results also show that the failure of WJs often develops in the HAZ material [46,67,68]. However, in the currently existing methods for assessing the strength of WJs, the HAZ is often omitted, and only the WM and the BM properties are taken into account [51,69], which leads to very conservative results during the analysis [52,53]. The presented results demonstrate the detrimental effect of the HAZ on the mechanical behavior of pipelines, including aged natural gas transit infrastructure.

To reflect the heterogeneity of WJ zones in the numerical model, the HAZ for the as-received WJ and the HAZ and the WM for the operated WJ were discretized into subzones with spatially varying material properties derived from experimental hardness profiles. This approach improves the fidelity of the stress–strain distribution compared to treating the HAZ and the WM as a single homogeneous zone. The analysis presented in this article develops a joint model that takes into account changes in the material’s strength and plasticity properties in the individual WM and HAZ subzones. The model was developed using precise HV10 hardness measurements, based on which subzones with similar hardness values were identified, as well as the correlation relationships between the hardness and the strength (*σ*_ys_r_, *σ*_uts_r_) and plasticity (*ε*_ys_r_, *ε*_uts_r_) of the steel, which were established and verified in previous studies [61]. Knowledge of the strength and plasticity characteristics has made it possible to define the constitutive stress–strain relationships in the form of an elastoplastic material with linear hardening for each subzone (layer) of the material in WJs.

The modeled joint element was subjected to a simulated tensile load in the direction of the pipe axis. High levels of loads (at or above the yield strength BM) were deliberately applied to induce plasticity in the WJs. It was found that the highest strain and stress levels occur in the HAZ layers on the BM side, which means that this area of the pipeline WJ is the most susceptible to the development of destructive processes. It should be noted that at a load slightly lower than the σ_ys_ of the BM, a local increase in strains was observed only in the HAZ directly at the BM.

The methodology for assessing the strength of welded elements presented in this paper has been verified on specimens containing WJs. They were made on steel of different strengths using various welding methods (Tungsten Inert Gas—TIG, Metal Inert Gas/Metal Active Gas—MIG/MAG and laser [46,60,61]). The verification was performed using the GOM Aramis 3D 5M video-optical system with digital image analysis [65].

Based on the results obtained, qualitative conclusions were drawn. However, the research methodology described allows us to perform quantitative assessments of the strength of welded elements for specific operating parameters. The methodology has combined zone-specific hardness-based constitutive modeling and finite element validation of heterogeneous WJs considering the effect of long-term operation of natural gas pipelines, which allows us to evaluate the mechanical response of aged pipeline weldments. It enables a more realistic prediction of the local mechanical behavior than approaches based on homogeneous material assumptions without taking into account the actual mechanical properties of aged material. It can be useful for assessing the possibility of repurposing existing natural gas pipelines for hydrogen transport, where the reliable assessment of aging effects and welded joint performance is important for resolving the issue. Therefore, the above findings contribute to pipeline safety assessment, aging infrastructure management, and the development of engineering tools supporting the transition to a hydrogen economy.

The results demonstrate that long-term service can lead to measurable changes in the local mechanical properties of WJs and highlight the importance of considering the heterogeneity of the BM, HAZ, and WM when assessing structural performance. The proposed hardness-based constitutive modeling approach offers an efficient methodology for characterizing these heterogeneous regions and can be applied to evaluate newly developed pipeline steels and welding procedures.

Furthermore, this study emphasizes that future pipeline materials should not only satisfy initial strength requirements but also maintain stable local mechanical properties throughout long-term service. This is particularly relevant for materials intended for hydrogen transport, where long-term degradation, weld integrity, and local mechanical heterogeneity may significantly influence structural performance. Consequently, the methodology presented in this work can support the development, optimization, and qualification of new pipeline steels and welding technologies by providing a framework for the assessment of local mechanical behavior and validation of numerical models used in structural integrity evaluations.

Welding-induced residual stresses are an important factor influencing the service performance of welded pipelines, which was not considered in this study. Although an increase in hardness was observed in certain regions of the WJs after 36 years of service, hardness measurements alone do not provide sufficient evidence to infer changes in the residual stress state. Residual stresses cannot be reliably quantified or correlated with hardness without dedicated experimental techniques. Furthermore, the residual stress distribution in operating pipelines evolves over time due to manufacturing processes, pressure loading, and other service-related effects, making its assessment a separate and complex research topic. Further studies will be focused on combining residual stress measurements with the proposed zone-specific constitutive modeling. The developed models can be used as a foundation for future investigations and modeling the effect of various important factors (fatigue behavior, fracture mechanisms, stress concentration, etc.) on failure prediction and the long-term structural integrity of aging welded pipelines, including those considered for hydrogen transport.

## 5. Conclusions

This article presents the results of experimental testing and finite element modeling of circumferential WJs in the X52 steel gas pipeline after 36 years of operation and in the as-received state.

The microstructure of the ferrite-pearlitic BM varies in its pearlite phase content, which affects the mechanical properties. The BM of the 36-year-old pipeline had higher hardness, yield strength and ultimate strength values compared to those of the as-received one.

The assessment of strength was based on the results obtained from a numerical simulation of tensile loading on the modeled joints. The obtained stress and strain distributions in individual zones of WJs made it possible to identify the most stressed one (heat-affected zone material) depending on the state (operated and as-received).

For the WJ of the 36-year-operated pipeline, the normalized graphs show higher relative extreme values of σ_11_/σ_ys_ = 1.25 compared to that for the as-received WJ, where σ_11_/σ_ys_ = 1.1. In the as-received WJ, strain concentration was observed for a stress load about 5% lower than the σ_ys_ of the BM, while for the operated WJ, a local strain increase was observed for a load even 11% lower than the σ_ys_ of the BM.

The finite element model was developed using precise HV10 hardness measurements, based on which subzones with similar hardness values were identified, and the correlation relationships between hardness and the strength and plasticity of the metal were established as well. The methodology for assessing the strength of welded elements presented in this paper has been verified on specimens containing WJs.

This study provides scientific guidelines for the development of an in situ non-destructive diagnostic procedure for long-distance gas pipelines, based on hardness measurements and the constitutive stress–strain relationships for each subzone of the material in WJs.

The methodology enables prediction of local mechanical properties of different WJ zones of aged pipelines and can be used for the assessment of the feasibility of repurposing existing pipeline infrastructure for hydrogen transport.

## Figures and Tables

**Table 1:** Strength and plasticity properties of individual zones of *WJ0*.

Welded Zone	HV 10	*σ*_uts_r_ (MPa)	*σ*_ys_r_ (MPa)	ε_uts_r_ (%)	ε_ys_r_ (%)
WM	230	735	579	10.71	0.38
HAZ_1	210	684	513	12.05	0.35
HAZ_2	185	623	429	13.95	0.30
HAZ_3	183	618	422	14.11	0.30
HAZ_4	181	613	416	14.28	0.29
BM	190	635	446	13.55	0.31

**Table 2:** Strength and plasticity properties of individual zones of *WJ36*.

Welded Zone	HV 10	*σ*_uts_r_ (MPa)	*σ*_ys_r_ (MPa)	ε_uts_r_ (%)	ε_ys_r_ (%)
WM_1	250	788	642	9.53	0.41
WM_2	240	761	611	10.10	0.40
WM_3	235	748	595	10.40	0.39
HAZ_1	230	735	579	10.71	0.38
HAZ_2	209	682	510	12.12	0.34
HAZ_3	195	647	463	13.16	0.32
BM	218	705	539	11.49	0.36

## Data Availability

The original contributions presented in the study are included in the article; further inquiries can be directed to the corresponding author.
